# Endoscopic diagnosis and prevalence of early gastric cancer in India: A prospective study

**DOI:** 10.1002/deo2.309

**Published:** 2023-11-04

**Authors:** Ashutosh Mohapatra, Sonmoon Mohapatra, Shruti Mahawar, Krushna Chandra Pani, Nachiketa Mohapatra, Mohan Ramchandani, Nageshwar Reddy, Mahesh K. Goenka, Noriya Uedo

**Affiliations:** ^1^ Department of Gastroenterology and Hepatology Sai Institute of Gastroenterology and Liver Sciences Bhubaneswar India; ^2^ Department of Gastroenterology and Hepatology Mayo Clinic Phoenix USA; ^3^ Department of Pathology Genx Diagnostic Center Bhubaneswar India; ^4^ Department of Gastroenterology and Hepatology Asian Institute of Gastroenterology Hyderabad India; ^5^ Department of Gastroenterology and Hepatology Apollo Gleneagles Hospitals Kolkata India; ^6^ Department of Gastroenterology and Hepatology Osaka International Cancer Institute Osaka Japan

**Keywords:** atrophic gastritis, early gastric cancer, gastric cancer, *Helicobacter pylori*, narrow band imaging

## Abstract

**Objectives:**

Although countries like Japan and South Korea have implemented nationwide endoscopic screening programs, there is limited evidence on the effectiveness of endoscopy for diagnosing early gastric cancer (EGC) in developing countries such as India. In the present study, we aimed to determine the feasibility of endoscopic detection of EGC from India.

**Methods:**

The data was prospectively collected for all patients ≥40 years who underwent a diagnostic upper endoscopy from April to September 2021. A single endoscopist who performed the endoscopic procedures completed 1‐month training in advanced endoscopy in Japan. Following the training, the endoscopist continued to engage in internet‐based discussions regarding his cases encountered. Prior to this training, the endoscopist had not detected any EGC cases during his 12‐year gastroenterology practice.

**Results:**

A total of 1033 patients were included in the study, with males accounting for 65.4% and a mean age 52 years. The average procedural time was 7.13 ± 4.8 min. A total of 25 patients (2.4%) were found to have GC, including 6 patients (0.6%) with EGC. Two patients had synchronous EGC lesions. All EGC patients were males, with an average age of 66 years. All EGCs were detected in the distal stomach in the presence of *Helicobacter pylori* infection and severe atrophic gastritis.

**Conclusion:**

Our findings showed that the endoscopic detection of EGC is feasible in India. Optimal training on endoscopic diagnosis of EGC can improve the detection of such lesion. Further studies are warranted to assess the optimization and implementation of an endoscopic screening program for EGC in India.

## INTRODUCTION

Gastric cancer (GC) is the fifth most common cancer globally with an estimated occurrence of 1.1 million new cases and 769,000 deaths (equivalent to one in every 13 deaths) in 2020, making it the fourth most common leading cause of death after lung, colorectum, and liver.^1^, ^2^ GC incidence rates are highest in Eastern Asia (Mongolia and Japan) and Eastern Europe, whereas lowest in Northern America, Northern Europe, and African regions.^2^ In India, GC remains the fifth most common cancer types, accounting for 7.2% of all cancer cases.^3^ Although the overall incidence of GC in India is lower compared to Eastern Asian countries, geographical variation exists, especially in southern and northeastern states of the country, where the incidence is comparable to the high‐incidence areas of the world.^4^, ^5^, ^6^

GC cases are often diagnosed late, resulting in a 5‐year survival rate of around 20% globally. However, in Japan and Korea, where population‐based screening programs exist, the survival rate for stages I and II GC exceeds 70%.^7^ One of the reasons for such difference is the identification of localized cancers through population‐based screening programs. Early detection enables curative endoscopic treatment, which is less invasive with a fewer complications compared to surgery. In India, the relatively low incidence of GC has created the perception that early GC (EGC) is extremely difficult or impractical to find, leading to the absence of clear screening guidelines. Although upper endoscopy is relatively inexpensive and is widely accessible at outpatient clinics or hospital settings in major cities, there is no published data on the effectiveness and feasibility of detecting EGC in the Indian population.

The objective of this study was to investigate the feasibility and rates of the endoscopic detection of EGC in Indian clinical practice.

## METHODS

### Endoscopic training

An Indian endoscopist (AM) underwent a comprehensive 1‐month training program in advanced endoscopy, at the Osaka International Cancer Institute in Japan in November 2019. The endoscopist had 12 years of experience in gastroenterology, conducting over 5000 gastroscopic examinations annually. Throughout the 12 years of practice, there had been no detection of EGC, resulting in a pretraining EGC detection rate of 0%.

The primary objectives of the onsite training in Japan encompassed several key aspects. First, it aimed to increase the awareness of EGC screening and enhance the ability to visually identify EGC during routine endoscopic examinations. This was achieved through daily discussions involving EGC cases, discussions of relevant research papers, and reviewing endoscopic videos. Second, the training provided insights into the quality of endoscopic examination using systemic stomach examination techniques. The factors highlighted for a thorough upper endoscopic examination included a good preparation using mucolytic agents to enhance the visibility of the gastric mucosa. Third, the training emphasized the use of magnifying endoscopy as a diagnostic tool for the characterization of detected EGC. Trainees completed 100 pictorial quiz questions to enhance knowledge and improve the accuracy of discriminating between neoplastic and non‐neoplastic lesions using magnifying narrow band imaging (NBI). Fourth, the training involved participating in hands‐on training with simulator‐ or animal‐models to acquire techniques for the diagnosis and treatment of EGC. Finally, effective communication between the endoscopist and pathologist was highlighted as an essential tool for standardizing pathological examinations of endoscopic submucosal dissection (ESD) specimens.

Upon returning to India, discussions regarding the endoscopic diagnosis of identified suspicious lesions were continued with a Japanese mentor, aiming to accumulate experience and to improve diagnostic ability. These discussions were facilitated through the use of an Internet messenger application (WhatsApp, Meta Platforms, Inc.). Additionally, the endoscopist attended webinar series and viewed web‐based videos as part of the ongoing training process.^8^

### Study design and settings

From April 2021 to September 2021, we conducted a prospective observational study in an outpatient setting at the Sai Institute of Gastroenterology and Liver Sciences located in Bhubaneswar, Odisha, Eastern India. The study protocol was approved by the Institutional Review Board ethics committee on March 12, 2021 (21‐0028). The sample size was determined according to the practicability of study conduction in an Indian clinical setting.

### Participants and endoscopic procedure

All individuals aged 40 years or older with abdominal symptoms were included in this study. Patients were instructed to fast from the morning of the procedure day but allowed to drink transparent liquid until 1 h before. Oral administration of a mixture of 100 mL water, 600 mg *N*‐acetylcysteine, and 0.5 mL (40 mg per mL) simethicone was done 15 min before the procedure. Sedation was not given for the procedures.

Video endoscopy systems used in this study were either EVIS LUCERA SPECTRUM or EVIS EXERA III (Olympus Medical Systems). A video endoscope (EVIS‐GIF‐H290Z, GIF‐H290EC, or GIF‐HQ190; Olympus) was used for high‐definition white light imaging and the NBI with zoom or near‐focus function. A black soft distal attachment (MAJ1989; Olympus) was used for magnification or near‐focus observation. Chromoendoscopy was not performed in this study.

All endoscopic examinations were conducted by a single endoscopist (AM). A systematic screening protocol for the stomach (SSS) was employed ensuring thorough examination and the elimination of blind spots.^9^ Efforts were made to remove mucous and froth from the mucosal surface. Air insufflation was used to distend the gastric wall for better EGC detection, especially at the greater curvature. At least 22 pictures were taken during each procedure according to the SSS protocol. Additionally, the gastric mucosa was inspected for high‐risk features, such as *Helicobacter pylori*–associated gastritis, endoscopic mucosal atrophy, or intestinal metaplasia where more time was dedicated to the search for EGC.

Suspicious lesions showing gastritis‐like, ulcerative, and polypoid appearance were meticulously searched in conventional white light imaging (C‐WLI).^10^ Especially, as the gastritis‐like lesions were the most challenging to detect, particular attention was paid to localized mucosal change of surface or color, spontaneous bleeding, or disappearance of vascular/mucosal pattern of the background mucosa.^11^ The suspicious lesions detected were examined with C‐WLI and magnifying or near‐focus NBI to differentiate between neoplastic and non‐neoplastic lesions. In magnifying or near‐focus NBI, the endoscopic diagnosis of EGC was made using the “VS (vessel plus surface) classification system” using two criteria: (1) well‐demarcated border and (2) an irregular microsurface or microvascular pattern.^10^, ^12^, ^13^

Biopsy specimens were taken from all suspicious lesions, were put in pods, and sent to the pathological department. Random biopsy for assessing histological gastritis was not performed in the study subjects.

### Pathological diagnosis

Histological diagnosis relied on hematoxylin and eosin staining. The expert gastrointestinal (GI) pathologist examined all specimens and made the histological diagnosis of GC, referring to both the WHO and the Japanese classification.^14^, ^15^

### Variables

Relevant clinical data, procedural information, and endoscopic and histological findings were recorded. The endoscopic procedural time was measured from scope insertion to withdrawal of the scope from the mouth. *H. pylori* infection was tested using rapid urease test or histology.^16^ A positive result in either test indicated *H. pylori* infection. *H. pylori* infection test was conducted for patients exhibiting endoscopic indications, such as erythema, erosions, and ulcerations, suggestive of the infection. However, for endoscopic diagnoses unrelated to *H. pylori* infection, such as esophagitis, esophageal candidiasis, or esophageal varices, this testing was not performed.

Endoscopic mucosal atrophy was evaluated using the Kimura–Takemoto classification, categorizing C‐I, C‐II as mild, C‐III, O‐I as moderate, and O‐II, O‐III as severe atrophy.^17^, ^18^ Descriptive findings of the stomach and gastric lesions were documented according to the Japanese classification of gastric carcinoma.^14^

### Study outcomes

EGC was defined as carcinoma confined to the mucosa and submucosa (T1), irrespective of the lymph node metastasis. On the other hand, advanced GC refers to carcinoma extending into the muscularis propria layer and beyond (≥T2), with or without confirmed nodal involvement.^14^

The primary objective was to prospectively evaluate the rate and characteristics of EGC in the Indian population. The secondary objective included assessing the rate of advanced GC, calculating the EGC ratio, and evaluating adverse events during the endoscopic procedures. EGC rate was defined as the number of newly diagnosed EGC cases divided by the total number of endoscopic procedures performed during the study period. EGC ratio was calculated as the ratio of newly detected EGCs to all newly detected GCs. Advanced GC rate was defined as the number of newly diagnosed advanced GCs divided by the total number of endoscopy procedures. Adverse events and severity were graded per the American Society for Gastrointestinal Endoscopy criteria.^19^

### Statistical analysis

Continuous variables were expressed as mean and standard deviation or median with interquartile range. Categorical variables were reported as frequency with percentage.

## RESULTS

The study included 1033 patients with a mean age of 52 ± 9 years, of whom 65.4% were males (Table 1). The most common indications for endoscopic procedures were dyspepsia (59.7%), chronic anemia (7%), and upper GI bleeding (5.5%). Among all, 1022 (98.9%) completed the SSS protocol, whereas 11 (1.1%) could not tolerate the procedure due to a lack of sedation. The mean procedural time was 7.1 ± 4.9 min. Out of a total of 1034 patients, 861 (83.3%) patients exhibited endoscopic atrophy. Among these, 392 (37.9%) had mild atrophy, 362 (35%) had moderate atrophy, and 107 (10.3%) displayed severe atrophy.

**TABLE 1:** Baseline characteristics of all included patients.

Patient demographics	*N* = 1033
Age at the time of presentation (years), mean (SD)	52 ± 9
Male, *n* (%)	677 (65.5)
Procedural indication, *n* (%)	
Dyspepsia	617 (59.7)
Anemia/upper GI bleed	73 (7.1)
Screening for esophageal varices in patients with chronic liver disease	57 (5.5)
Others	291 (28.2)
Dietary history/potential risk factors, *n* (%)	
Alcohol	21 (2)
Smoking	20 (1.9)
Chewing tobacco	126 (12.2)
Salted fish	138 (13.4)
Procedural characteristics	
Procedural time in min, mean (SD)	7.1 ± 4.9
Intolerance due to lack of sedation, *n* (%)	11 (1.1)
Endoscopic mucosal atrophy according to Kimura–Takemoto classification, *n* (%)	
None	172 (16.7)
Mild (C‐I, C‐II)	392 (37.9)
Moderate (C‐III, O‐I)	362 (35.0)
Severe (O‐II, O‐III)	107 (10.3)
Main endoscopic findings, *n* (%)	
Early gastric cancer	6 (0.6)
Advanced gastric cancer	19 (1.8)
Gastric erosion	438 (42.4)
Benign gastric ulcer	50 (4.8)
Gastric xanthoma	26 (2.5)
Gastric polyps	56 (5.4)
Esophageal varices	16 (1.5)
Normal examination	172 (16.6)
Others (such as esophageal cancer, esophagitis, and candidiasis)	250 (24.2)
Number with *Helicobacter pylori* positive, *n* (%)	487/546 (89.2)

### Primary outcome

**FIGURE 1:**
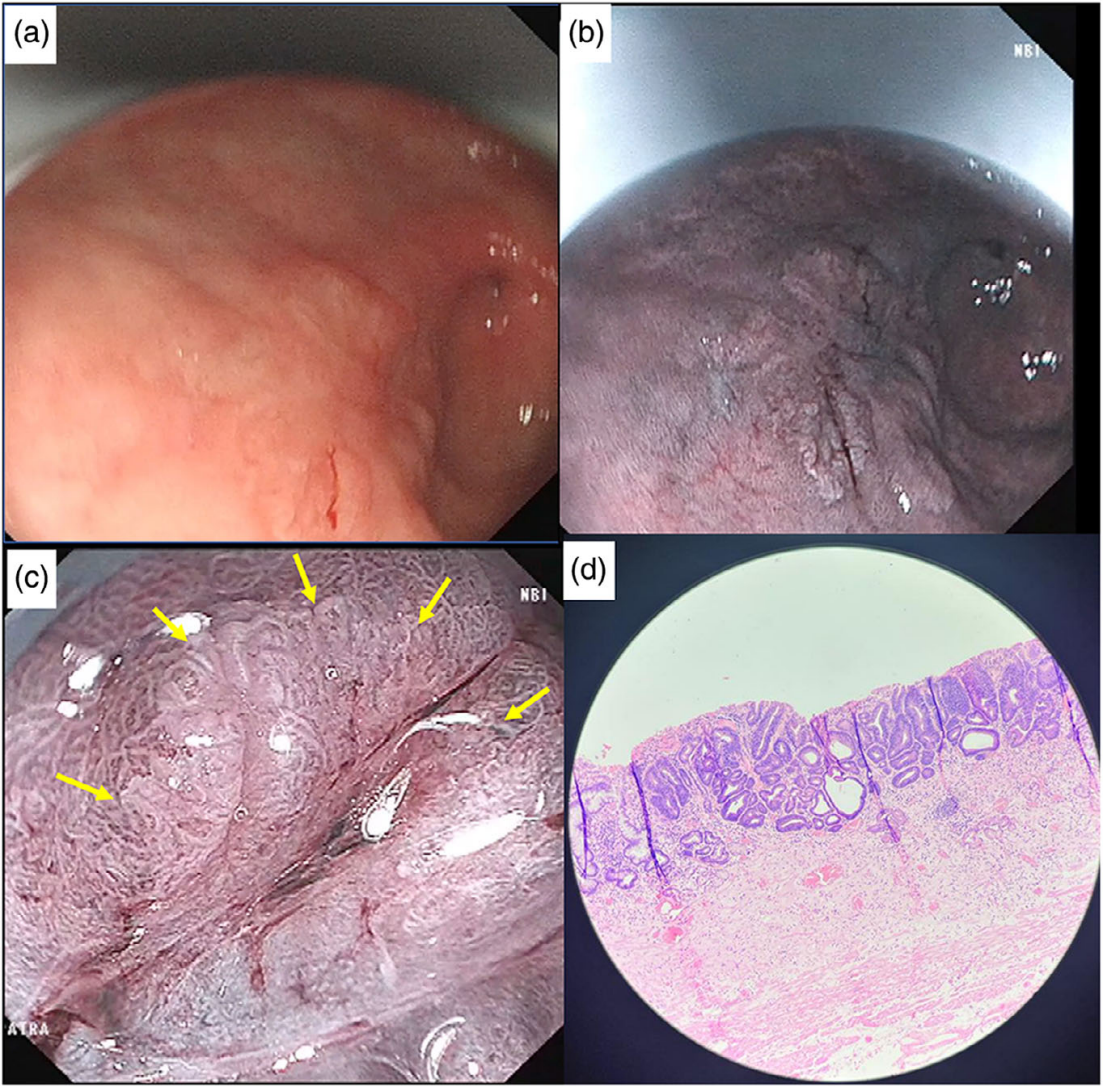
Conventional white light imaging (a) and narrow band imaging (NBI) (b) of a 20 mm superficial depressed type lesion in the anterior wall of antrum (Patient 1). Spontaneous bleeding was seen with water irrigation. Magnified narrow band imaging showed irregular microsurface pattern with demarcation line (c). Histopathology demonstrated dysplastic gastric glands (well differentiated) consisted of neoplastic cells having vesicular to hyperchromatic nucleus, small nucleolus, and eosinophilic cytoplasm, infiltrating the lamina propria. Deep and lateral margins were negative of the tumor. There was no lymphovascular invasion seen (d).

**FIGURE 2:**
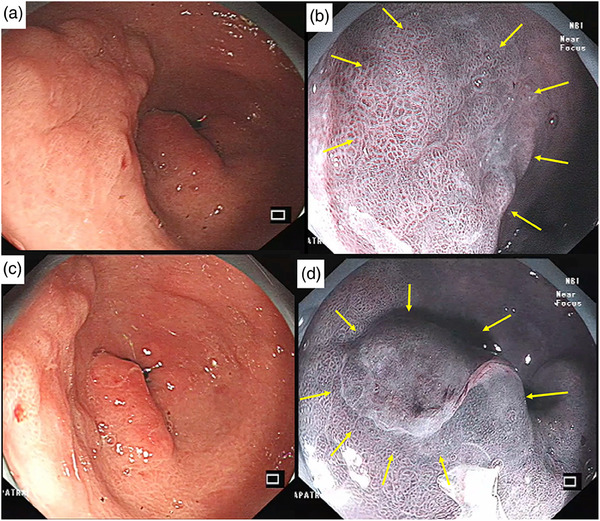
In conventional white light imaging (C‐WLI), a 10 mm superficial depressed type lesion was found in the anterior wall of antrum in the background of atrophic gastritis (Patient 2) (a). There was a demarcation line (arrows) with irregular microsurface pattern (b). Conventional white light imaging of a 8 mm reddish superficial elevated synchronous lesion at the pylorus (c). With near‐focus observation, the microsurface pattern was irregular. Demarcation line was seen (arrows, d). Histopathology of the endoscopic submucosal dissection specimen showed well‐differentiated tumor limited to lamina propria with negative lateral and deep margins. There was no lymphovascular invasion identified.

**FIGURE 3:**
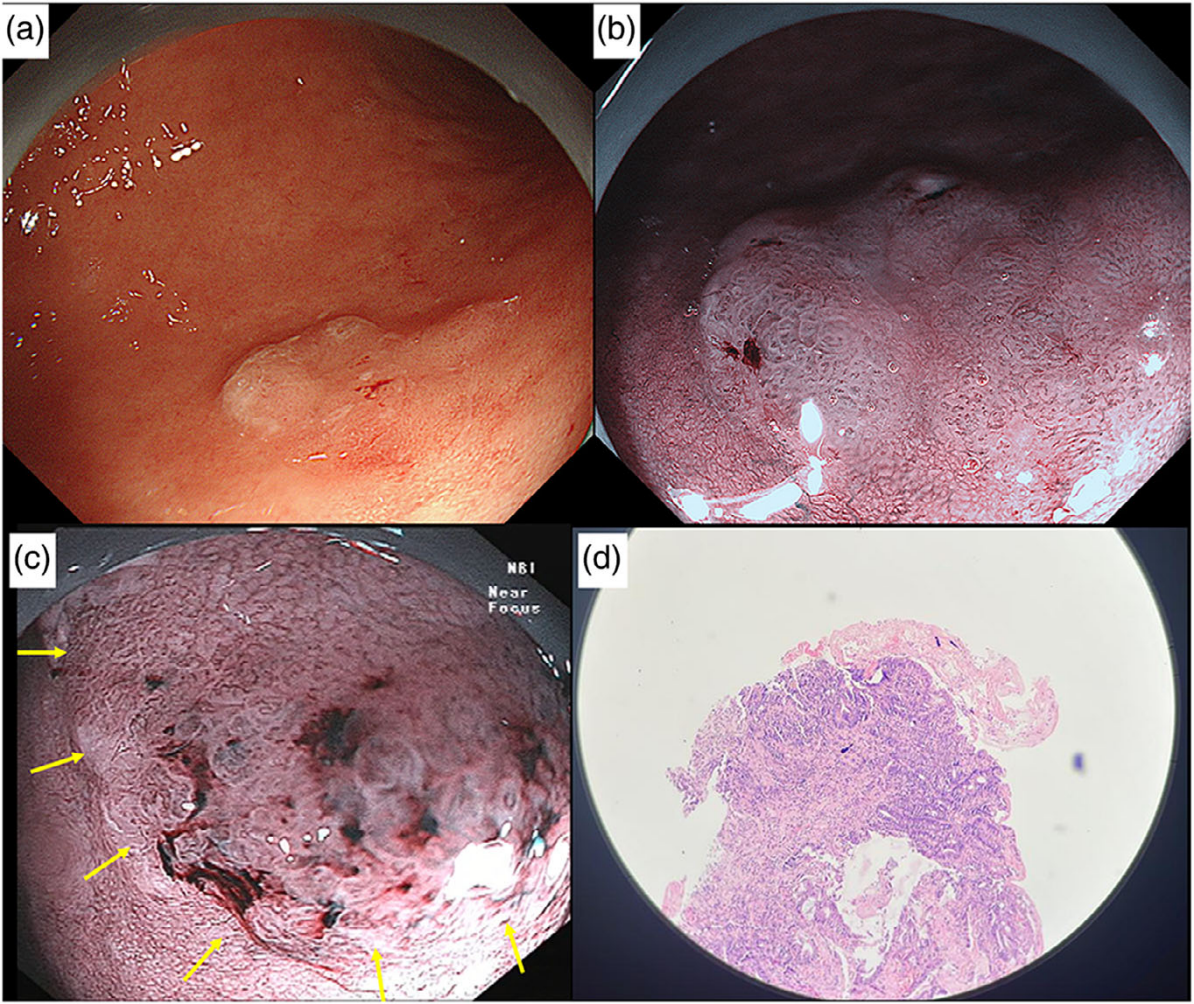
Conventional white light imaging (a) and narrow band imaging (b) showed a 10 mm superficial elevated lesion in the posterior wall of the gastric body (Patient 3). The lesion had a demarcation line with irregular microvessel pattern (c). Pathology showed 0.9 × 0.6 × 0.2 cm^3^ tumor infiltrating the muscularis mucosae. Deep and lateral margins were free of tumor. There was no lymphovascular invasion seen.

**FIGURE 4:**
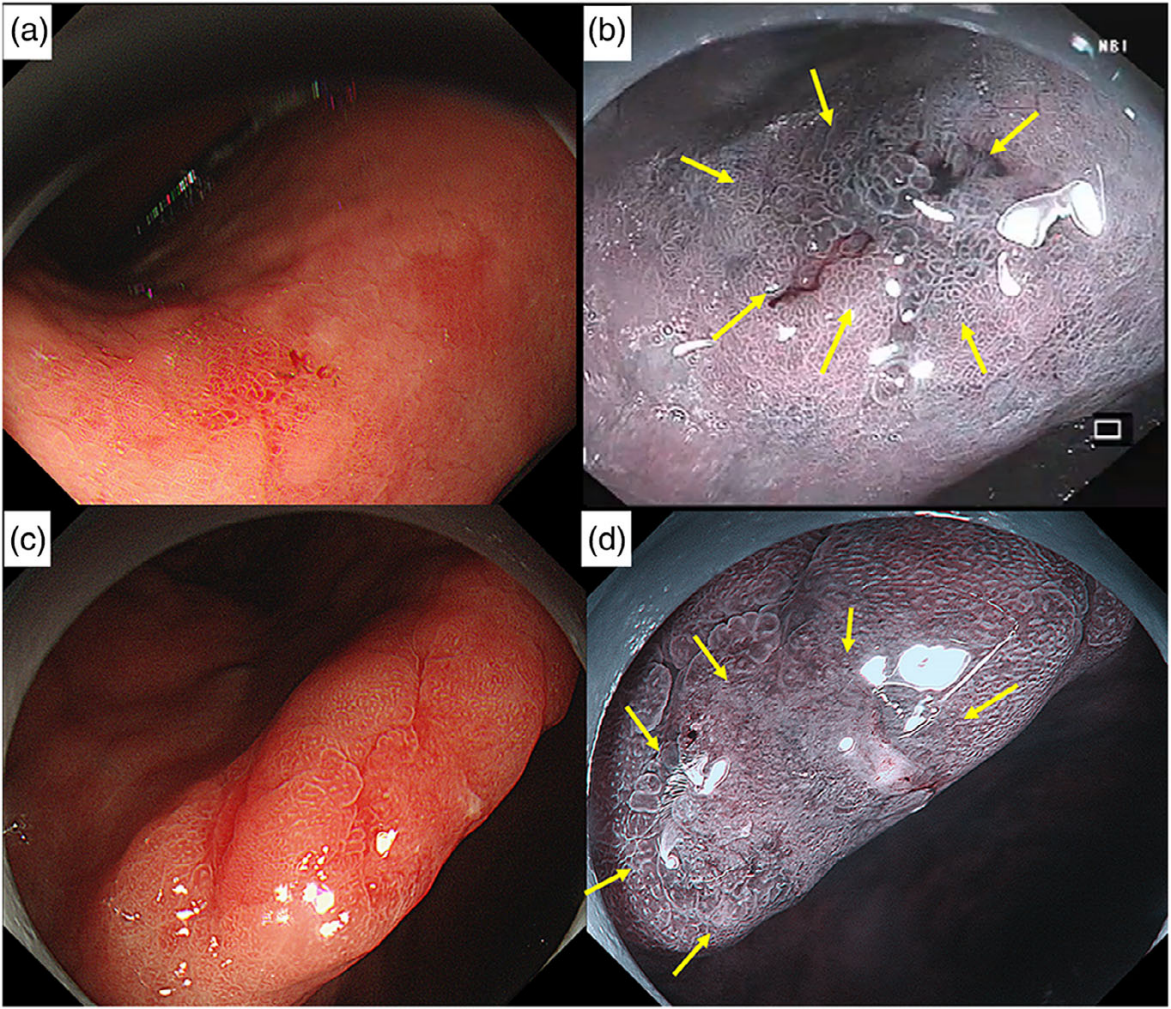
Conventional white light imaging showed a 7 mm reddish superficial depressed type of lesion in the lesser curvature of the lower corpus (Patient 4) (a). The extent of the lesion was more clearly seen, represented as irregular surface pattern with demarcation line in narrow band imaging (b). Unfortunately, this patient was lost to follow‐up. A 8 mm reddish superficial depressed type lesion was recognized at the incisura angularis (Patient 5) (c). Irregular microvessels with clear demarcation line seen with magnified narrow band imaging (d). Endoscopic submucosal dissection was performed, and pathology demonstrated well‐differentiated lesion limited to the lamina propria. The lateral and deep margins were negative and no lymphovascular invasion identified.

**FIGURE 5:**
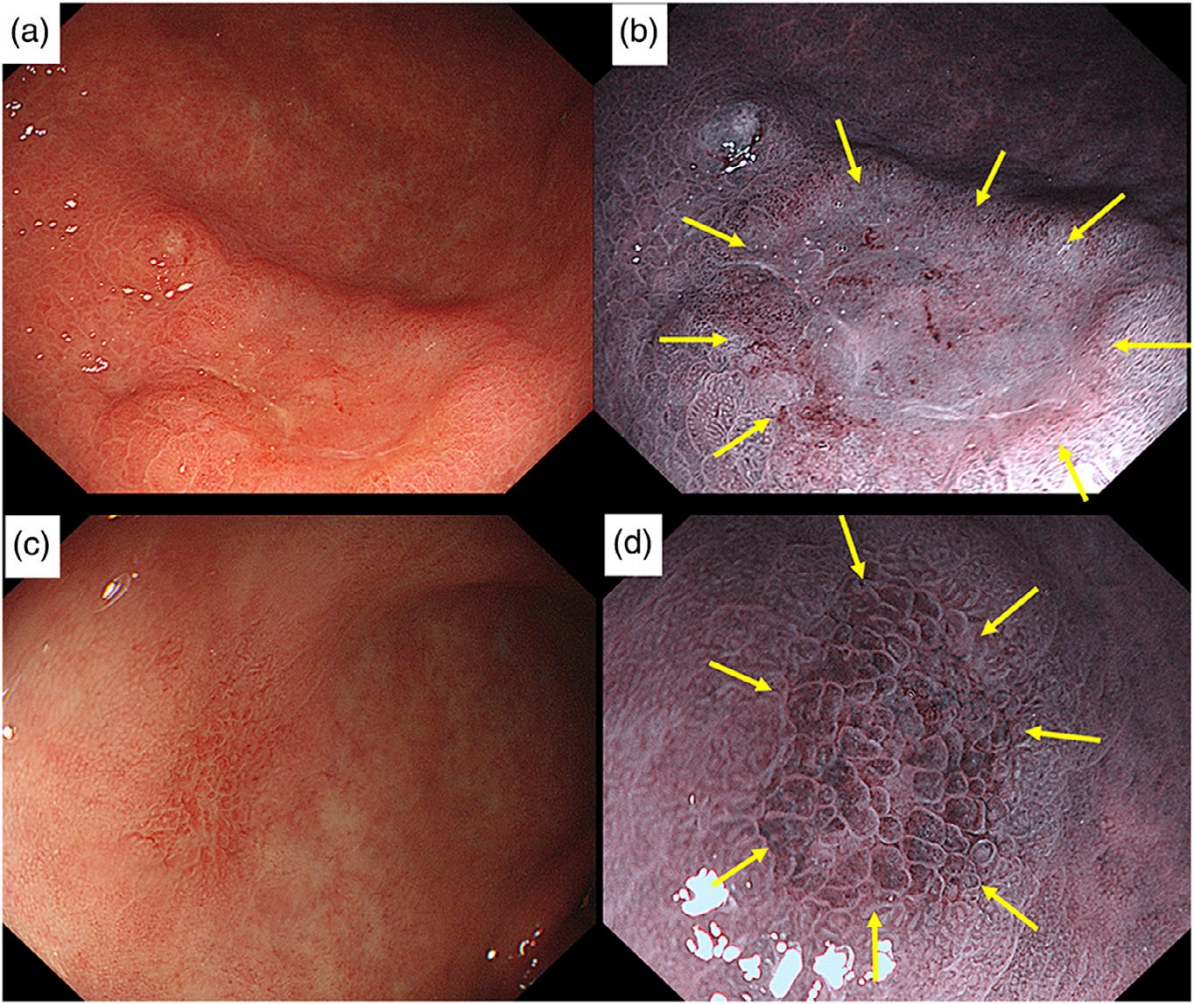
A 12 mm superficial depressed type lesion in the greater curvature of the lower corpus (a) (Patient 6). In narrow band imaging, well‐demarcated border was contrasted, showing irregular vessel pattern and absent surface pattern (b). Histopathology showed well‐differentiated adenocarcinoma invading lamina propria. A 8 mm reddened superficial flat type synchronous lesion was noted in the anterior wall of the angularis (c). Irregular microsurface with clear demarcation seen under narrow band imaging (d). Endoscopic submucosal dissection specimen showed well‐differentiated adenocarcinoma invading muscularis mucosa. The lateral and deep margins of both the lesions were free of tumor. There was no evidence of lymphovascular invasion or perineural invasion seen.

Among the 1033 patients, 6 (0.6%) were diagnosed with EGC, all of whom were males with an average age of 66 ± 3.9 (range: 60–72) years. Two patients had synchronous lesions. All EGCs were located in the distal stomach, associated with *H. pylori* infection and severe atrophic gastritis. Out of the six patients with EGC, five underwent ESD, resulting in successful curative resection, whereas one patient did not receive any treatment and was lost to follow‐up. Details regarding lesion characteristics, treatment, and histopathology are summarized in Figures 1, 2, 3, 4, 5 and Table 2.

**TABLE 2:** Risk factors and lesion characteristics of the patients with early gastric cancer.

						C‐WLI findings		M‐ or NF‐NBI findings				Histopathology			
Lesions	Age/Sex	Lesion size (mm)	Location	Risk factors	Endoscopic atrophy	Color	Morphology	Demarcation line	Microsurface pattern	Microvessel pattern	Treatment	Differentiation	Depth of invasion	Margin status	LVI
1.	67/M	20	L	*Helicobacter pylori* +	Severe	Isochromatic	0‐IIc	Present	Irregular	Absent	ESD	Well‐differentiated	Lamina propria	Negative	Absent
2.	67/M	10	L	*H. pylori* +	Severe	Isochromatic	0‐IIc	Present	Irregular	Irregular	ESD	Well‐differentiated	Lamina propria	Negative	Absent
3.	67/M	8	L	*H. pylori* +	Severe	Reddish	0‐IIa	Present	Irregular	Absent	ESD	Well‐differentiated	Lamina propria	Negative	Absent
4.	72/M	10	L	*H. pylori* +	Severe	Pale	0‐IIa	Present	Irregular	Irregular	ESD	Well‐differentiated	Muscularis mucosa	Negative	Absent
5.	66/M	7	L	*H. pylori* + Alcohol +	Severe	Reddish	0‐IIb	Present	Irregular	Regular	None (lost f/u)	–	–	–	–
6.	64/M	8	L	*H. pylori* +	Severe	Reddish	0‐IIa+IIc	Present	Irregular	Irregular	ESD	Well‐differentiated	Lamina propria	Negative	Absent
7.	60/M	10	L	*H. pylori* +	Severe	Isochromatic	0‐IIc+IIa	Present	Absent	Irregular	ESD	Well‐differentiated	Muscularis mucosa	Negative	Absent
8.	60 M	8	L	*H. pylori* +	Severe	Isochromatic	0‐IIb	Present	Irregular	Irregular	ESD	Well‐differentiated	Lamina propria	Negative	Absent

Abbreviations: C‐WLI, conventional white light imaging; ESD, endoscopic submucosal dissection; f/u, follow‐up; L, lower distal body; LVI, lymphovascular invasion; M‐NBI, magnified narrow band imaging; NF‐NBI, near‐focus narrow band imaging.

### Secondary outcome

Out of the total patients, 25 (2.4%) were diagnosed with GC, including the six EGC cases. Besides 6 patients with EGC, 19 patients (1.8%) were diagnosed with advanced GC, resulting in an EGC detection ratio of 24% during the study period.

No adverse events were reported during any of the endoscopic procedures.

## DISCUSSION

To the best of our knowledge, this study is the first to assess the feasibility and rate of EGC detection in Indian clinical practice. In Eastern India, we found that GC exists; in fact, 2.4% of patients undergoing diagnostic endoscopy were diagnosed with GC. This can be attributed to the high prevalence of *H. pylori* infection and chronic atrophic gastritis in Indian population. In well‐trained endoscopist's eyes, the prevalence of EGC was 0.6%, accounting for 24% of all GC. The lesion characteristics were comparable to those described in Japanese studies, primarily of the superficial “gastritis‐like” type, often seen in patients with *H. pylori* infection.

EGC poses a diagnostic challenge as it often presents as subtle mucosal findings that can be easily overlooked without a thorough examination of the stomach. Several studies have performed in assessing the rate of interval EGC after a negative endoscopy and have reported a missed cancer rate of 2.3%–40%.^20^, ^21^, ^22^, ^23^, ^24^ A meta‐analysis, including 10 studies, have found pooled miss rates of upper GI cancers of 6.4% within 1 year and 11.3% within 3 years before diagnosis.^25^ A comprehensive understanding of typical endoscopic and pathologic findings is necessary for the successful diagnosis of EGC. In other words, even if an adequate amount of time is spent in the stomach, an endoscopist cannot identify EGC without having a proper knowledge of how to diagnose it. Suspicion of EGC is often raised by common endoscopic findings, such as color change (i.e., red or pale), loss of vascularity, slight elevation or depression, nodularity or mucosal thickening, and abnormal convergence or flattening of folds. Recognizing common endoscopic abnormalities and distinguishing neoplastic from non‐neoplastic lesions are crucial in diagnosing EGC. Our study suggested that proper on‐site and online training supervised by Japanese endoscopists proved beneficial in improving knowledge and endoscopic practice in EGC detection.

Detecting EGC in India faces challenges due to the varied incidence of GC in different regions and the low rate of localized stage diagnosis. Moreover, limited awareness and healthcare access contribute to underestimating the true incidence, particularly in rural areas where many deaths occur without medical attention.^5^ There are several other factors that are likely associated with the underdiagnosis of EGC in India. First, the training in gastroenterology lacks uniformity and mainly focuses on the improvement of technical skills and the recognition of major pathologies. Moreover, for the technical skill, trainers pay attention more to endoscopic maneuvers such as insertion or retroflexion of the endoscope rather than emphasis on how to properly examine the upper GI tract.^26^ Accordingly, physicians without postgraduate training in gastroenterology can perform routine endoscopic procedures by attending short, nonstructured training courses, which may result in inadequate training with a potential to miss major diagnoses.^27^ Second, endoscopists in India typically spend only a few minutes on upper GI examination, and protocols like the SSS Protocol are rarely followed.^28^ Third, sedation is not commonly used due to cost and high patient volume and mucolytics^29^ or defoamers are rarely used, despite their potential benefits. Finally, proper documentation, including the patient information, technical details, findings, endoscopic therapies, and image documentations, is not commonly practiced. Handwritten reports with missing details are still prevalent, and there is a lack of standardized quality metrics for routine endoscopic procedures.

Prior to the training, the rate of EGC detection was 0%. This can be attributed to our limited knowledge of how to diagnose EGC. We did not have high‐definition endoscopes, and our approach lacked adherence to a systemic screening protocol. Our upper endoscopic examinations were brief with limited utilization of mucolytics. During the on‐site training in Japan, the endoscopist observed live demonstrations of EGC diagnosis, attended educational lectures, and participated in quality endoscopy sessions. The training also provided insights into systemic stomach examination techniques and the benefits of routine use of magnified endoscopy and VS classification. Subsequently, the online training continued, including attending webinar series, watching web‐based videos, and actively participating by fielding questions and engaging with the mentor on WhatsApp.

The training had a significant impact on shifting endoscopy practices in India. The endoscopist successfully identified eight EGC lesions (0.6%) in 6 months in India; the detection rate aligns with finding from a previously published study from China (EGC detection rate in a trained group of 0.7%).^30^ We believe this was mainly achieved by the acquisition of knowledge and awareness in detecting these lesions rather than the use of advanced imaging technology such as NBI. In fact, all EGC lesions were detected in C‐WLI observation. Furthermore, it is noteworthy that the diagnostic yield of magnifying endoscopy in detecting EGC was limited, as its primary role was to characterize already detected lesions with the non‐magnified C‐WLI. The use of internet‐based learning systems, as well as receiving valuable feedback from Japanese experts regarding lesion characteristics, played a crucial role in expanding knowledge regarding endoscopic detection. To facilitate continuous self‐assessment and feedback, all our procedures were video recorded, leading to improved endoscopic diagnosis capabilities. Furthermore, several factors, including the use of mucolytics, high‐definition endoscope, distal attachment cap, and implementation of SSS protocol, contributed to enhancing the diagnosis of EGC.

Given the high incidence of *H. pylori* infection in India (∼80%),^31^ our practice has also observed a high prevalence of *H. pylori*–associated chronic atrophic gastritis, intestinal metaplasia, and low‐grade dysplasia, which are often overlooked endoscopically. In our EGC patients, all had endoscopic mucosal atrophy in C‐WLI and intestinal metaplasia in NBI. Recognizing these high‐risk conditions and performing careful inspections, biopsies, and surveillance endoscopy in patients with high‐risk mucosa significantly improved EGC detection.

This study has several limitations. First, it was conducted in a single center in an outpatient clinic in a major city in Eastern India, so the generalizability of the findings to other parts of the country needs to be validated. Second, all endoscopic procedures were carried out without sedation, which may have caused intolerance in some patients, increasing the likelihood of missing lesions. Although there was no direct evidence to support the use of sedation to enhance the detection of EGC, the use of sedation improves patient acceptance for surveillance endoscopy as well. Third, the procedures were performed only on symptomatic patients, so the rate may not reflect the true prevalence of EGC in the population. Therefore, the necessity and cost‐effectiveness of conducting population‐based GC screening in high‐prevalence states in India are unknown.

In summary, our study suggests that by adhering to a standardized endoscopic protocol, the detection of EGC is feasible in India. The study highlights the necessity for a change in endoscopic practice to enhance the diagnosis and treatment of EGC, and further large‐scale studies are warranted to assess the optimization and implementation of an endoscopic screening program for EGC in India.

## CONFLICT OF INTEREST STATEMENT

None.
